# Hospital Needs and Invested Funds

**Published:** 1906-12-29

**Authors:** 


### HOSPITAL NEEDS AND INVESTED FUNDS.

This question is not generally understood by hospital governors. In "Burdett's Hospitals and Charities for 1900" (Chapter I.) and for 1901 (Chapter IV.) the question is fully gone into, and anyone interested in the subject will find there complete information covering the whole ground. We have laid it down as a rule which is now generally accepted that where a hospital has invested funds which exceed a sum equal to five times its average total expenditure, any money standing to the credit of capital account at such an institution may properly be utilised for the purposes of new buildings or the reconstruction of the existing accommodation. A voluntary hospital should be dependent in the interests of the patients and of the public upon the free-will offerings of the people for whose benefit it exists. Under such a system the efficiency of the management must always continue high, for otherwise the public in each generation is not likely to continue to support it. So inefficiency would come to mean ultimate decadence and failure. The East Suffolk and Ipswich Hospital has placed before the governors a building scheme which will entail an expenditure of some £12,000. Towards this sum £5,000 has been promised. At the general meeting held recently a governor, Mr. F. Long, suggested that the balance of £7,000 should be taken from the capital account. In our view Mr. Long's suggestion is one which may properly be adopted, seeing that the invested funds of this hospital amount to £66,000 and that the average expenditure is about £8,000 a year. We hope, therefore, that the governors may determine (1) to proceed with the reconstruction and new buildings at once, and (2) to sell out capital to provide the difference in cost between the sum specially given for this purpose and the total expenditure entailed. We have no doubt the adoption of this course will result in the Committee being placed in a position, within the next few years, to replace the sum thus taken from capital. Large and generous givers believe in a hospital being managed upon business principles. For this reason they would probably give liberally to the investment fund as a testimony of their approval of the wisdom displayed by the management in adopting the course here suggested.

